# Pentagalloyl glucose suppresses MSU crystal–induced gout inflammation and arachidonic acid production *in vitro* and *in vivo*

**DOI:** 10.3389/fphar.2026.1812913

**Published:** 2026-04-24

**Authors:** Sadiq Umar, Yu Lu, Sugasini Dhavamani, Poorna C. R. Yalagala, Mateusz S. Wietecha, Sriram Ravindran

**Affiliations:** 1 Department of Oral Biology, University of Illinois Chicago, Chicago, IL, United States; 2 Endocrinology, Diabetes and Metabolism, Department of medicine, University of Illinois Chicago, Chicago, IL, United States

**Keywords:** arachidonic acid pathway, inflammation, macrophages, MSU-gout model, pentagalloyl glucose

## Abstract

**Background:**

Gout is an acute inflammatory arthritis triggered by monosodium urate (MSU) crystal deposition and activation of innate immune responses. In addition to inflammasome signaling, emerging evidence suggests that metabolic reprogramming of arachidonic acid (AA) pathways amplifies inflammatory responses during gout flares. However, the contribution of upstream fatty acid desaturation processes that regulate endogenous AA availability remains poorly defined. 1,2,3,4,6-Penta-O-galloyl-β-D-glucose (PGG) is a naturally occurring polyphenol with reported anti-inflammatory activity, but its effects on MSU-induced fatty acid metabolism and gouty inflammation have not been well established.

**Methods:**

Publicly available bulk and single-cell transcriptomic datasets from human and mouse gout studies were analyzed to assess dysregulation of AA-associated pathways. MSU-induced inflammatory responses were examined in mouse bone marrow–derived macrophages and in a murine MSU-induced gout model. Macrophages were treated with PGG prior to MSU stimulation, and inflammatory cytokine production, phagocytosis, and expression of fatty acid desaturases were assessed. Lipidomic analysis of macrophages and plasma was performed using gas chromatography–mass spectrometry (GC–MS) to quantify arachidonic acid and related fatty acids. *In vivo* disease severity, cytokine expression, and anti-inflammatory markers were evaluated following PGG treatment.

**Results:**

Analysis of public datasets revealed consistent dysregulation of arachidonic acid–associated inflammatory pathways during gout flares. In macrophages, MSU stimulation increased expression of fatty acid desaturases FADS1 and FADS2 and promoted accumulation of arachidonic acid, concomitant with robust production of pro-inflammatory cytokines. PGG treatment significantly suppressed MSU-induced FADS1, FADS2 and arachidonic acid levels, and attenuated pro-inflammatory cytokine production. PGG also markedly impaired macrophage phagocytosis of MSU crystals. *In vivo*, PGG treatment significantly reduced clinical disease severity in an MSU-induced gout model, suppressed fatty acid desaturation and arachidonic acid accumulation in plasma, decreased pro-inflammatory cytokine expression, and enhanced anti-inflammatory markers.

**Conclusion:**

These findings identify fatty acid desaturation as an important metabolic contributor to gouty inflammation and demonstrate that PGG suppresses MSU-induced inflammation by limiting endogenous arachidonic acid availability, reducing inflammatory amplification, and impairing MSU crystal phagocytosis. Targeting upstream fatty acid metabolism represents a potential therapeutic strategy for modulating acute gout flares beyond conventional anti-inflammatory approaches.

## Introduction

Gout is a prevalent and increasingly common form of inflammatory arthritis, with a rising global incidence in both developed and developing countries (FitzGerald et al., 2020; Dalbeth et al., 2016; Dalbeth et al., 2010; Richette and Dalbeth, 2024). The initiation of gouty inflammation occurs when resident joint macrophages phagocytose monosodium urate (MSU) crystals. Crystal uptake activates innate immune signaling pathways, notably through Toll-like receptors (TLR2 and TLR4), leading to NF-κB activation NF-κB (Ma et al., 2018; Lu et al., 2024; Chung et al., 2016; Chen et al., 2019; Singh et al., 2019; So and Martinon, 2017) and assembly of the NLRP3 inflammasome (Chen et al., 2019; Lian et al., 2021; Renaudin et al., 2020; Wu et al., 2022). This cascade promotes the release of pro-inflammatory cytokines and drives the recruitment of neutrophils to the inflamed joint. Activated neutrophils further amplify inflammation by releasing reactive oxygen species, proteases, cytokines, chemokines, and lipid mediators (Lu et al., 2024; Ling et al., 2024; de Lima et al., 2023; Silva et al., 2023; Crisan et al., 2016; Sirisinha, 2011; McCormack et al., 2009; Jia et al., 2022; Shan et al., 2021; Yang et al., 2026).

MSU crystals potently stimulate arachidonic acid (AA) metabolism, resulting in robust prostanoid production—particularly prostaglandin E_2_ (PGE_2_)—through enhanced cytosolic phospholipase A_2_ (cPLA_2_) activity and cyclooxygenase-2 (COX-2) expression in immune cells and osteoblasts. Elevated levels of prostanoids within gouty synovial fluid are strongly associated with the cardinal clinical features of acute gout flares, including severe pain, swelling, and erythema (Bouchard et al., 2002; Korotkova and Jakobsson, 2014; Margalit et al., 1997). Despite the established role of AA-derived lipid mediators in inflammatory diseases, their specific contribution to gout pathogenesis remains incompletely understood. AA metabolism generates a diverse array of bioactive lipids, including prostaglandins, thromboxanes, and leukotrienes, which are potent regulators of immune cell activation and resolution (Zhang et al., 2020; Zhao et al., 2025). Recent studies have demonstrated that MSU crystals upregulate PTGS2 (COX-2) expression in peripheral monocytes from patients with advanced gout, linking AA metabolism to systemic inflammatory burden (Gu et al., 2023). In addition, MSU-induced leukotriene production by neutrophils and elevated synovial fluid levels of leukotriene B_4_ (LTB_4_) further support a pathogenic role for lipid mediators in gout, exceeding those observed in other inflammatory arthritides such as rheumatoid arthritis (Filgueiras et al., 2015).

Current treatment of acute gout relies largely on NSAIDs, colchicine, and corticosteroids, which effectively suppress symptoms but are often limited by systemic toxicity and contraindications in patients with renal, cardiovascular, or metabolic comorbidities (Richette and Dalbeth, 2024; Cipolletta et al., 2026; Umar et al., 2025). NSAIDs primarily inhibit prostaglandin synthesis via cyclooxygenase blockade, while colchicine reduces inflammation by impairing microtubule-dependent neutrophil recruitment; however, neither strategy directly regulates upstream arachidonic acid metabolism or coordinated lipid mediator signaling during MSU-driven inflammation.

1,2,3,4,6-Penta-O-galloyl-β-D-glucose (PGG) is a naturally occurring polyphenolic compound that has been reported to exhibit broad anti-inflammatory, anti-oxidative, and immunomodulatory activities across multiple experimental systems. Previous studies have demonstrated that PGG suppresses inflammatory mediator production in macrophages, inhibits oxidative stress, and attenuates tissue injury in models of inflammatory and metabolic disease. Mechanistically, PGG has been shown to interfere with upstream innate immune signaling and lipid metabolic pathways rather than acting solely on terminal cytokine effectors, suggesting a capacity to modulate inflammatory amplification at an early stage. Despite these observations, the therapeutic potential of PGG in acute gouty inflammation and its impact on monosodium urate (MSU)–driven arachidonic acid metabolism remain poorly defined. In the present study, we aimed to delineate the anti-inflammatory mechanisms of PGG in macrophages and to evaluate its therapeutic efficacy in a murine MSU-induced gout model, with particular emphasis on its regulation of arachidonic acid–derived prostanoid production and downstream inflammatory responses.

## Methods

### Gene expression analysis from gout patients and mouse model

Bulk RNA sequencing dataset GSE242872 submitted by Yin et al. (2024) for gout model at 8 and 24 h and GSE191054 Human macrophages activated with MSU (Cobo et al., 2022) and single-cell RNA sequencing dataset GSE211783 submitted by Yu et al. (2024) to evaluate the expression of arachidonic pathway.

### Myeloid cells

Mouse bone marrow derived macrophages (mBMMs) were isolated from 8-week C57BL/6J mice. Mouse bone marrow cells were cultured with mouse M-CSF (20 ng/mL) for 3 days to obtain myeloid cells differentiated *in vitro* as MΦs (10% FBS/DMEM). On day 4, MΦs were pretreated for 18 h with DMSO (PBS), PGG (5 μM, Sigma #G7548, dose is based on our previous studies) (Umar et al., 2022; Panipinto et al., 2025) in serum free RPMI. Thereafter cells were stimulated with MSU (Singh et al., 2019; Umar et al., 2025) (100 μg/mL; Sigma #U2875) for 24 h. For running ELISA (Protein) and qRT-PCR (mRNA) analysis. Cell viability assay was done in BMDMs with increasing concentrations of PGG (0.1–10 µM) for 24 h. Cell viability was assessed using the MTT assay according to manufacturer instructions (G3580, Promega).

### Real-time RT-PCR

RNA isolated using Trizol and was reverse transcribed to cDNA using the RevertAid RT Reverse Transcription Kit (Thermo Scientific). SYBR green gene expression master mix (Bio-Rad) to perform qRT-PCR. Data was normalized with GAPDH and are presented as fold changes in RNA levels compared to control treatment, calculated following the 2^−ΔΔCT^ method.

### ELISA for cytokine analyses

Conditioned media from the macrophage, pretreated with PGG (5 µM) overnight followed by stimulation with MSU for 24 h was collected and cytokine levels of IL-1β, IL-6, TNF-α and IL-18 were measured using DuoSet ELISA (enzyme-linked immunosorbent assay) kits (R&D Systems, MN).

### *In vitro* phagocytosis assay

The phagocytic activity of macrophages was assessed using the Vybrant™ Phagocytosis Assay Kit (Life Technologies™). Briefly, macrophages (1 × 10^4^) were seeded in a 96-well flat-bottom plate, pretreated with PGG overnight, and stimulated with MSU for 2 h. The culture medium was then replaced with 100 µL of the prepared fluorescent Bioparticle suspension, followed by incubation at 37 °C for 2 h. After incubation, the Bioparticle suspension was removed, and the cells were washed twice with PBS. Subsequently, 100 µL of prepared Trypan Blue suspension was added, incubated for 1 min, and the fluorescence intensity was measured using a plate reader with ∼480 nm excitation and ∼520 nm emission, following the manufacturer’s instructions.

### Lipid extraction and fatty acid analysis by GC/MS

Blood was collected by cardiac puncture into heparinized syringes, and plasma was isolated by centrifugation at 1,500 × g for 15 min at 4 °C. Total lipids were extracted from plasma using a modified version of a previously published method (Sugasini et al., 2017). Briefly, 100 µL of plasma or macrophages lysate (*in vitro*) were mixed with 800 µL of 50% methanol in water containing 0.01 N HCl, followed by the addition of 2 mL chloroform. Samples were vortexed for 30 s, 1 mL water was added, and samples were vortexed again for 30 s before centrifugation. The lower chloroform phase was collected, dried under nitrogen, and used for fatty acid analysis. Lipid extracts were converted to fatty acid methyl esters (FAMEs) as previously described (Sugasini et al., 2017). Dried lipids were resuspended in 0.5 mL toluene containing 25 µg of 22:3 free fatty acid as an internal standard and 250 µg butylated hydroxytoluene. Methanolic HCl (0.3 mL of 8% HCl in methanol) was added, and samples were heated at 100 °C for 1 h under nitrogen. The reaction was neutralized with 1.0 mL of 0.33 N NaOH, and FAMEs were extracted twice with 3 mL hexane. Combined hexane extracts were dried under nitrogen, reconstituted in 30 µL hexane, and 1 µL was injected into the GC/MS system. Fatty acid analysis was performed using a Shimadzu QP2010SE GC/MS equipped with a Supelco Omegawax capillary column (30 m × 0.25 mm × 0.25 µm), with data acquired over a total ion current range of m/z 50–400.

### Murine model of gout

All animal studies were approved by UIC Animal Care and Use Committee (protocol # 2024-042). After 7 days of acclimatization, 8-10-weeks-old male C57BL/6J mice (Jackson Laboratory) were divided into three groups; a) Control b) Monosodium Uric acid-MSU (gout Model) c) MSU + PGG, (n = 5). In the treatment group, PGG (25 mg/kg, daily oral gavage) was administered from day 0. C57BL/6 mice at 8–10 weeks are susceptible to the development of gouty arthritis when injected with MSU crystal (0.5 mg) suspended in 25 µL endotoxin free PBS or PBS control will be injected into footpad of mice anaesthetized with 2.5%–4% isoflurane. This model is one of the most synchronized and reliable rodent models of gout and produces the least distress (Singh et al., 2019; Wu et al., 2022; Joosten et al., 2016; Crisan et al., 2017; Cheng et al., 2022; Lan et al., 2021; Zhang et al., 2018). The Δ ankle circumferences of both the hind ankles from each animal were averaged and monitored for clinical signs of inflammation.

### Statistical analysis

For comparison between multiple groups, one-way ANOVA followed by Tukey’s or Šídák’s multiple comparison test was done using Graph Pad Prism10 software. Values of *p* < 0.05 were considered significant.

## Results

### Altered arachidonic acid–associated metabolic pathways are linked to gout

**FIGURE 1:**
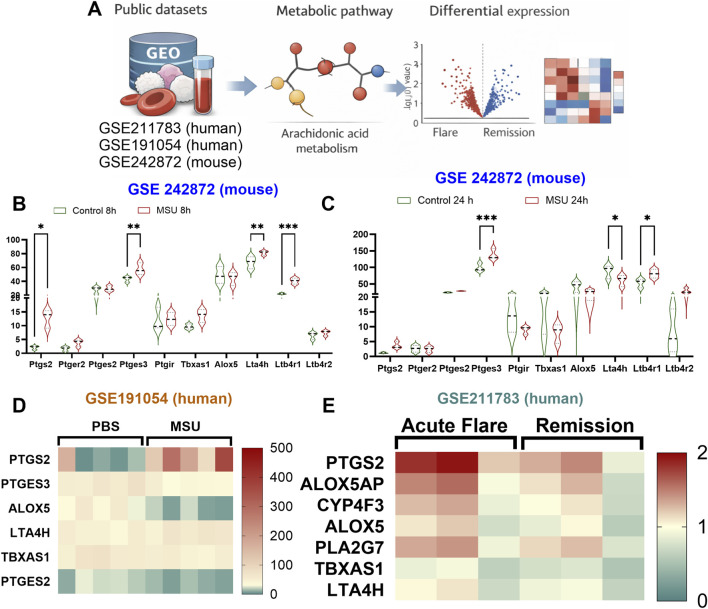
Arachidonic acid is associated with the severity of gout. **(A)** Schematic diagram for analysis of public data in mouse and human **(B–E)** Expression levels of Arachidonic acid related genes in gout.

Arachidonic acid (AA) metabolism has emerged as an important amplifier of crystal-induced inflammation. To explore the involvement of AA-associated pathways in gout, we analyzed publicly available bulk and single-cell transcriptomic datasets derived from MSU-induced mouse models and human gout samples. Across datasets, genes involved in prostanoid and leukotriene pathways (e.g., PTGES, ALOX5, and LTA4H) were consistently dysregulated during gout flares compared with remission or control conditions, highlighting a conserved activation of AA-derived inflammatory programs (Figure 1).

These analyses support the concept that AA metabolism contributes to gout pathogenesis across species and disease stages and provide a rationale for targeting upstream metabolic processes that regulate AA availability during MSU-driven inflammation (Yu et al., 2024).

### PGG suppresses MSU-induced fatty acid desaturation and arachidonic acid accumulation in macrophages

**FIGURE 2:**
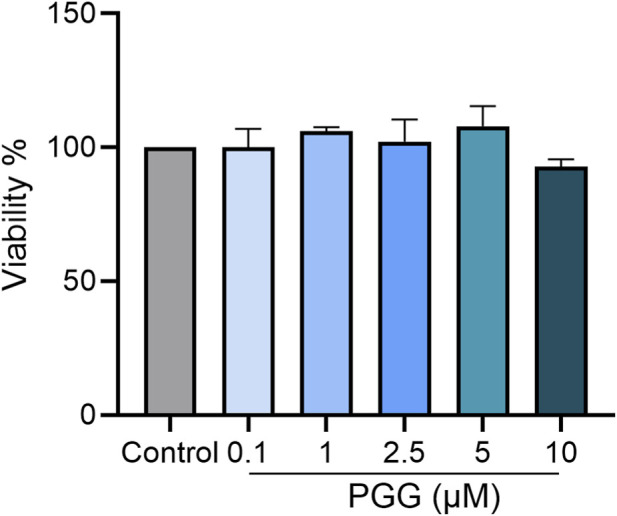
PGG does not affect macrophage viability at experimental concentrations. Bone marrow–derived macrophages (BMDMs) were treated with increasing concentrations of pentagalloyl glucose (PGG; 0.1–10 µM) for 24 h. Cell viability was assessed using the MTT assay according to the manufacturer’s instructions. Absorbance was measured at 570 nm and normalized to untreated control cells.

We assessed macrophage viability following PGG treatment to exclude the possibility that the observed anti-inflammatory effects were due to cytotoxicity, PGG did not significantly affect BMDM viability at concentrations up to 5 µM (Figure 2), confirming that the inhibitory effects observed in this study are not attributable to cell toxicity.

**FIGURE 3:**
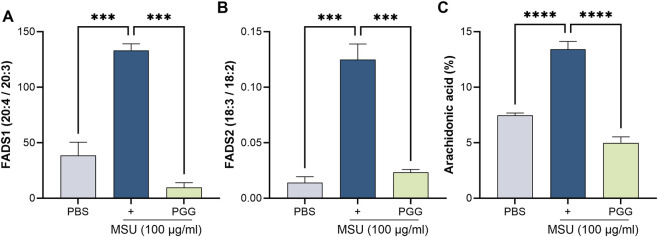
GC–MS profiling of Fatty acid in macrophages. Total lipids were extracted, and fatty acids were derivatized to fatty acid methyl esters (FAMEs) and quantified by gas chromatography–mass spectrometry (GC–MS). Fatty acids associated with FADS2 (Δ6-desaturase) activity, including linoleic acid–derived intermediates, and FADS1 (Δ5-desaturase) products, and arachidonic acid. (n = 3) **(A–C)** The data are shown as mean ± SEM *** represents p < 0.001. Significant differences were determined by one-way ANOVA following Šídák’s multiple comparison test.

Given the central role of AA as a substrate for inflammatory lipid mediators, we next investigated whether PGG modulates endogenous AA biosynthesis in macrophages. MSU stimulation significantly increased the expression of FADS2 and FADS1, the Δ6- and Δ5-desaturases that catalyze the conversion of linoleic acid to AA. Consistent with enhanced fatty acid desaturation, GC–MS analysis revealed a marked accumulation of AA in MSU-stimulated macrophages. PGG treatment significantly attenuated MSU-induced FADS1, FADS2 and reduced AA levels (Figure 3). These results indicate that PGG suppresses MSU-induced fatty acid desaturation, thereby limiting intracellular AA availability.

### PGG attenuates MSU-induced inflammatory cytokine production

**FIGURE 4:**
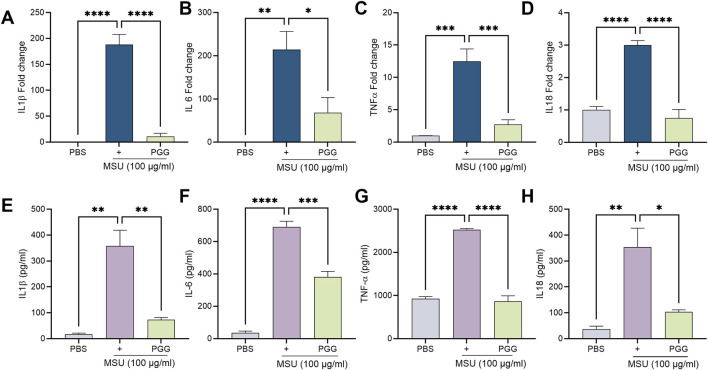
PGG suppresses MSU-induced pro-inflammatory cytokine production and gene expression in macrophages. Monocytes were isolated from mouse bone marrow and differentiated into macrophages. On day 4, cells were pretreated overnight with PGG (5 µM) and subsequently stimulated with MSU crystals (100 μg/mL) for 24 h. **(A–D)** Cells were harvested for RNA isolation, and mRNA expression of IL-1β, TNF-α, IL-6, and IL-18 was assessed by real-time RT-PCR. **(E–H)** Conditioned media were collected and analyzed by ELISA to quantify the secretion of IL-1β, TNF-α, IL-6, and IL-18. Data are presented as mean ± SEM (n = 4). Statistical significance was determined by one-way ANOVA followed by Šídák’s multiple-comparison test. *p < 0.05, **p < 0.01, ***p < 0.001, ****p < 0.0001.

Increased AA availability amplifies inflammatory responses by fueling the generation of bioactive lipid mediators that potentiate cytokine production. Concomitant with FADS1/2 inhibition, PGG robustly attenuated MSU-induced cytokine production. MSU exposure induced pronounced secretion of IL-1β, IL-6, IL18 and TNF-α, while PGG treatment significantly reduced these cytokines, demonstrating broad suppression of the inflammatory response (Figures 4A–D). At the transcriptional level, MSU stimulation strongly upregulated IL-1β, IL6, IL18 and TNF-α expression, all of which were significantly downregulated following PGG treatment, consistent with reduced inflammatory activation (Figures 4E–H). Together, these findings indicate that PGG reduced AA accumulation concomitant with reduced cytokine production in macrophages.

### PGG treatment disrupts MSU-induced phagocytosis

**FIGURE 5:**
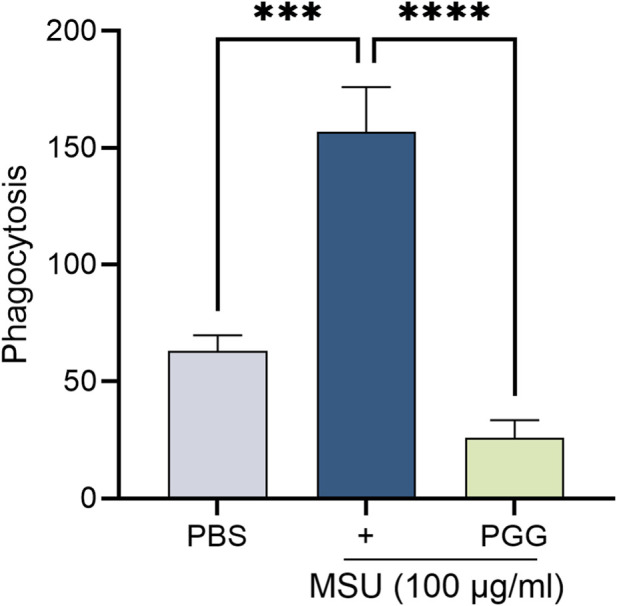
PGG impairs MSU induced phagocytosis. Macrophages were seeded into 96 well plate and incubated overnight with PGG (5 µM) and stimulated with MSU (100 μg/mL) for 2 h and absorbance was taken following manufacturer instructions. n = 3. The data are shown as mean ± SEM, *** represents p < 0.001 and **** denotes p < 0.0001. Significant differences were determined by one-way ANOVA following Šídák’s multiple comparison test.

Phagocytosis represents a critical early event in gout pathogenesis. Macrophage uptake of monosodium urate (MSU) crystals initiates innate immune activation and downstream inflammatory processes. Our results show that PGG treatment significantly reduced MSU crystal phagocytosis in macrophage (Figure 5), indicating that PGG directly interferes with cellular mechanisms required for MSU uptake.

### PGG reduces gout severity by modulating fatty acid metabolism and inflammatory responses *in vivo*

**FIGURE 6:**
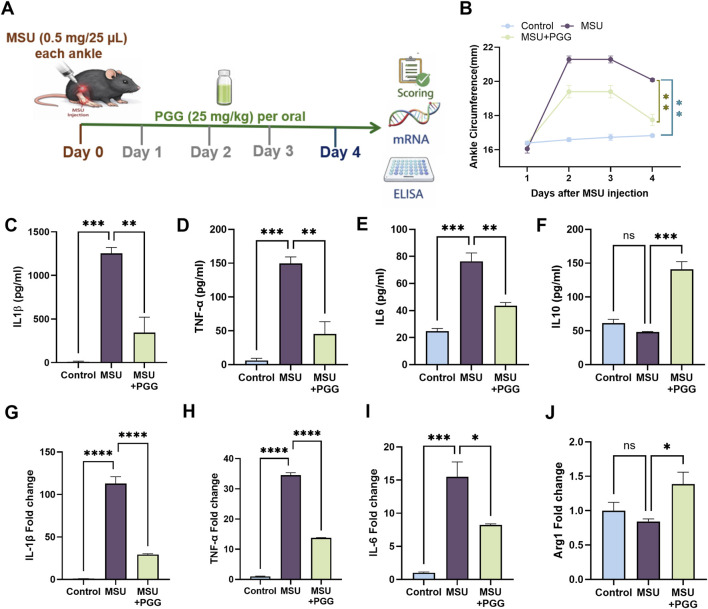
PGG attenuates MSU-induced gouty inflammation by suppressing scoring and inflammatory cytokines *in vivo*. **(A)** Mice were subjected to an MSU-induced gout model and treated with PGG as indicated. **(B)** Clinical gout severity scores over the experimental period show significant attenuation of MSU-induced disease following PGG treatment. **(C–F)** Protein levels of IL-1β, IL-6, and TNF-α in joint tissues were markedly increased after MSU challenge and significantly reduced by PGG, whereas IL-10 levels were enhanced. **(G–J)** qPCR analysis of joint tissues demonstrates that MSU stimulation upregulated IL-1β, IL6 and TNF-α expression, all of which were significantly suppressed by PGG treatment, while Arg1 expression was significantly increased. Data are presented as mean ± SEM (n = 4). Statistical significance was determined by one-way ANOVA followed by Šídák’s multiple-comparison test. *p < 0.05, **p < 0.01, ***p < 0.001, ****p < 0.0001.

To evaluate the therapeutic efficacy of PGG *in vivo*, an MSU-induced gout model was established in mice (Figure 6A). MSU administration resulted in a marked increase in clinical disease severity, as reflected by significantly elevated gout scores compared with controls. PGG treatment significantly attenuated MSU-induced disease severity, leading to a pronounced reduction in clinical scoring over the course of the experiment (Figure 6B).

At the protein level, MSU challenge induced robust production of IL-1β, IL-6, and TNF-α in joint tissues, whereas PGG treatment significantly suppressed these pro-inflammatory cytokines. In contrast, IL-10 levels were significantly increased in PGG-treated mice, indicating a shift toward an anti-inflammatory environment (Figures 6C–F).

Consistent with these findings, transcriptional analysis revealed that MSU stimulation markedly upregulated IL-1β, IL-6 and TNF-α expression, all of which were significantly reduced following PGG treatment (Figures 6G–J). Conversely, Arg1 expression was significantly enhanced in PGG-treated mice, supporting the induction of an anti-inflammatory phenotype.

**FIGURE 7:**
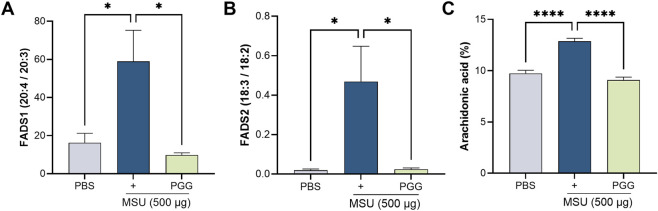
*In vivo* plasma GC–MS profiling of Fatty acid. Total lipids were extracted from mouse plasma and converted to fatty acid methyl esters (FAMEs) for quantification by gas chromatography–mass spectrometry (GC–MS). Fatty acids reflecting FADS2 (Δ6-desaturase) activity, including linoleic acid–derived intermediates, and FADS1 (Δ5-desaturase) products, including arachidonic acid, were quantified **(A–C)**. Data are presented as mean ± SEM (n = 5). Statistical significance was determined by one-way ANOVA followed by Šídák’s multiple-comparison test. *p < 0.05, ****p < 0.0001.

To further assess whether PGG modulates fatty acid metabolism *in vivo*, expression of key fatty acid desaturases was examined in plasma from MSU-induced gout mice. MSU challenge significantly increased FADS1, FADS2 and arachidonic acid, consistent with enhanced arachidonic acid biosynthetic activity during gouty inflammation. Notably, PGG treatment markedly suppressed MSU-induced FADS1, FADS2, and AA indicating inhibition of fatty acid desaturation pathways *in vivo* (Figures 7A–C).

Together, these findings indicate that PGG suppresses MSU-induced gouty inflammation *in vivo* by inhibiting fatty acid metabolism, reducing pro-inflammatory mediators, and promoting an anti-inflammatory gene expression profile.

## Discussion

Gout is increasingly recognized as a metabolically driven inflammatory disease in which monosodium urate (MSU) crystals engage innate immune cells and reprogram lipid metabolism to amplify inflammatory responses. In the present study, we demonstrate that PGG exerts potent anti-gout activity both *in vitro* and *in vivo* by targeting fatty acid desaturation pathways, suppressing MSU crystal phagocytosis, and shifting macrophage responses toward an anti-inflammatory phenotype.

A key finding of this work is the identification of fatty acid desaturases FADS1 and FADS2 as regulated targets during gouty inflammation. These enzymes catalyze critical steps in the biosynthesis of arachidonic acid (AA), a central substrate for pro-inflammatory lipid mediators. Our *in vitro* data show that MSU stimulation induces FADS1 and FADS2 expressions in macrophages, consistent with metabolic priming toward enhanced AA availability. Importantly, PGG significantly suppressed FADS1 and FADS2 expression, indicating that PGG interferes with upstream lipid metabolic reprogramming rather than solely blocking downstream inflammatory outputs. These findings extend prior observations that AA metabolism contributes to gout pathogenesis and position fatty acid desaturation as a previously underappreciated regulatory node in MSU-driven inflammation.

Beyond metabolic regulation, our data highlight phagocytosis as a critical functional target of PGG. Macrophage uptake of MSU crystals is an essential initiating event in gout, triggering intracellular signaling cascades and perpetuating tissue inflammation (de Almeida et al., 2022; Xu et al., 2023; Baggio et al., 2021; Bousoik et al., 2020; Piao et al., 2022). We observed that PGG markedly reduced MSU crystal phagocytosis in macrophages, suggesting that modulation of membrane lipid composition or cytoskeletal dynamics may underlie its inhibitory effects. Given that fatty acid composition directly influences membrane fluidity and phagocytic capacity, suppression of FADS1/FADS2-driven lipid remodeling provides a plausible mechanistic link between altered metabolism and reduced MSU uptake.

The *in vivo* relevance of these findings was confirmed in an MSU-induced mouse model of gout. PGG treatment significantly reduced clinical disease severity, demonstrating robust therapeutic efficacy. PGG suppressed MSU-induced expression of Fads1 and Fads2 in serum, validating that fatty acid metabolic regulation occurs *in vivo* and is not restricted to cell culture systems. Concomitantly, PGG reduced pro-inflammatory mediators at both the protein and transcriptional levels, including IL-1β, IL-6, TNF-α, while enhancing IL-10 and Arg1 expression. This coordinated molecular shift supports a model in which PGG not only dampens inflammatory activation but also actively promotes resolution-associated macrophage programs.

Notably, the increase in IL-10 and Arg1 suggests that PGG favors a reparative immune environment rather than inducing broad immunosuppression. This is particularly relevant in gout, where excessive inflammation coexists with cycles of spontaneous resolution. By limiting fatty acid–driven amplification loops and promoting anti-inflammatory gene expression, PGG may help restore immune balance within the inflamed joint.

Collectively, these findings support a multilevel mechanism of action for PGG in gout: (i) inhibition of FADS1/FADS2-mediated fatty acid desaturation, (ii) attenuation of MSU crystal phagocytosis, and (iii) suppressing MSU induced the inflammatory responses in macrophage. This integrated mechanism distinguishes PGG from conventional anti-inflammatory strategies that primarily target single cytokines and underscores the therapeutic potential of metabolic modulation in crystal-induced inflammatory diseases.

In summary, our study identifies fatty acid desaturation as a critical contributor to gout pathogenesis and establishes PGG as a metabolically active anti-inflammatory agent capable of suppressing MSU-induced inflammation *in vitro* and *in vivo*. These findings provide a strong rationale for further development of PGG or related metabolic modulators as disease-modifying therapies for gout.

### Limitations

Although our findings show that PGG suppresses FADS1/2 expression and reduces arachidonic acid accumulation during MSU-induced inflammation, the present study establishes an association rather than direct enzymatic regulation of FADS activity. Future studies using genetic or enzymatic approaches will be needed to determine whether PGG directly targets FADS enzymes or upstream metabolic pathways. In addition, although oral PGG showed significant anti-inflammatory efficacy in the MSU gout model, its pharmacokinetic properties, including bioavailability and metabolic conversion, remain incompletely defined. Previous studies indicate that polyphenolic compounds such as PGG may undergo metabolic transformation while retaining biological activity (Jiamboonsri et al., 2015; Patnaik et al., 2019; Krook and Hagerman, 2012). Finally, the acute MSU model used here does not fully recapitulate the complexity of chronic hyperuricemia and recurrent gout flares observed clinically, and will be consider in further studies.

## Data Availability

The original contributions presented in the study are included in the article/supplementary material, further inquiries can be directed to the corresponding authors.
